# Primary Sjögren’s syndrome: new perspectives on salivary gland epithelial cells

**DOI:** 10.1186/s40001-024-01967-5

**Published:** 2024-07-17

**Authors:** Jiaqi Hou, Yiyi Feng, Zhixia Yang, Yimei Ding, Dandan Cheng, Zhonghao Shi, Rouxin Li, Luan Xue

**Affiliations:** 1 Rheumatology Department, Yueyang Hospital of Integrative Medicine, Shanghai University of Traditional Chinese Medicine, 110 Ganhe Road, Hongkou District, Shanghai, 200437 China; 2 Shanghai Skin Diseases Hospital, 200 Wuyi Road, Changning District, Shanghai, 200050 China

**Keywords:** Primary Sjögren’s syndrome, Salivary gland epithelial cells, Function, Structure, Pathogenesis

## Abstract

Primary Sjögren’s syndrome (pSS) is a chronic autoimmune disease primarily affecting exocrine glands such as the salivary glands, leading to impaired secretion and sicca symptoms. As the mainstay of salivation, salivary gland epithelial cells (SGECs) have an important role in the pathology of pSS. Emerging evidence suggests that the interplay between immunological factors and SGECs may not be the initial trigger or the sole mechanism responsible for xerostomia in pSS, challenging conventional perceptions. To deepen our understanding, current research regarding SGECs in pSS was reviewed. Among the extensive aberrations in cellular architecture and function, this review highlighted certain alterations of SGECs that were identified to occur independently of or in absence of lymphocytic infiltration. In particular, some of these alterations may serve as upstream factors of immuno-inflammatory responses. These findings underscore the significance of introspecting the pathogenesis of pSS and developing interventions targeting SGECs in the early stages of the disease.

**Graphical Abstract:**

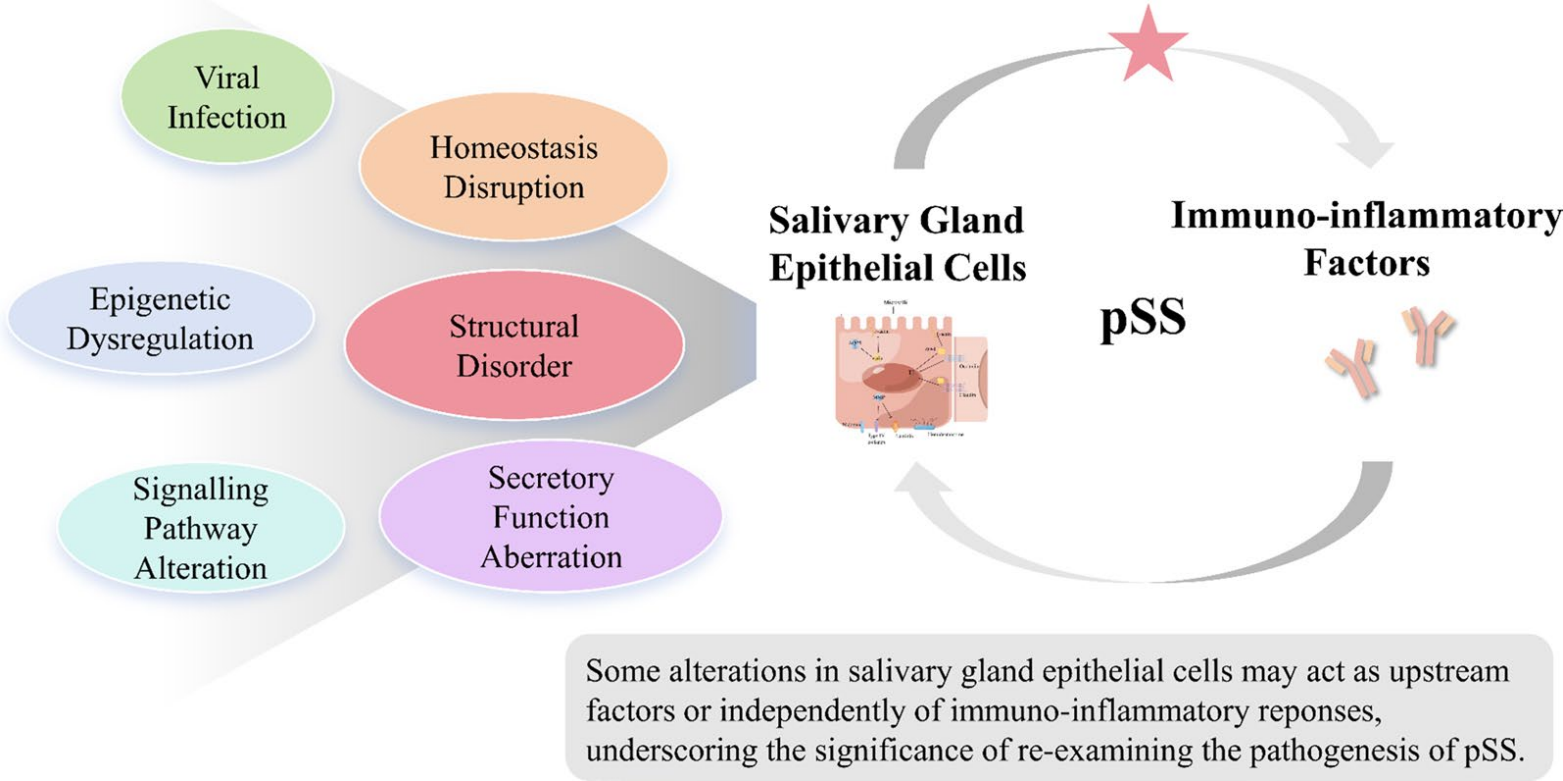

## Introduction

Primary Sjögren’s syndrome (pSS) is a prevalent chronic autoimmune disease characterized by lymphocytic infiltration in exocrine glands, primarily involving the lacrimal and salivary glands. The majority of pSS patients suffer from dryness, pain and fatigue, while 30–40% may develop systemic manifestations over time. Compared to the general population, pSS patients face a substantially increased risk of lymphoma, which poses a threat to their overall survival [1, 2].

Xerostomia is a hallmark symptom of pSS, affecting more than 90% of the patients [3, 4]. Salivary flow in pSS patients is notably reduced compared to those only with sicca symptoms [5]. Inadequate salivary secretion can further cause burning sensation in the mouth, oral infections, frequent ulcers, and rampant caries, ultimately resulting in difficulties with swallowing or speaking. These challenges tremendously diminish patients’ quality of life [6, 7].

Unfortunately, there is scant robust evidence hitherto indicating that current immunological interventions for pSS effectively alleviate oral dryness. Regarding conventional synthetic disease-modifying antirheumatic drugs (csDMARDs), studies have shown limited efficacy of the mainstream treatment hydroxychloroquine in managing pSS [8–10]. Other csDMARDs such as methotrexate [11], cyclosporine [12] and leflunomide [13] have also demonstrated minimal improvement in objective secretory function. In terms of biologics, tumor necrosis factor inhibitors [14, 15], anti-CD20 agent rituximab [16–20], B-cell activating factor inhibitor belimumab [21–23], the selective T-cell co-stimulation modulator abatacept and the CD40 inhibitor [24, 25] have yielded similarly unsatisfactory results in enhancing salivary secretion.

The disappointing efficacy of these interventions may be attributed to accumulating evidence suggesting that progression of the disease and lymphocytic infiltration do not necessarily parallel secretory dysfunction [26–30]. Studies have demonstrated no significant difference in focus score between pSS patients without dryness and those experiencing extreme dryness [31]. Similarly, regardless of the presence of germinal centers, which indicate more severe lymphocyte aggregation in salivary glands (SGs), salivary secretion did not differ significantly [32].

Epithelial cells, as the central players in the pathogenesis of autoimmune diseases, have garnered significant attention from researchers in recent years. Some scholars propose that both pSS and primary biliary cholangitis (PBC) fall under the spectrum of generalized autoimmune epithelitis [33–36]. Both diseases predominantly affect peri-menopausal women and have a frequent coexistence in clinical practice. Histologically, these two conditions are characterized by infiltration of B and T cells affecting exocrine glands and biliary epithelium, respectively. There is a certain degree of serological overlap between the two diseases, with detectable antinuclear antibodies, anti-Ro-52 antibody, and anti-centromere antibody. Immunosuppressive therapy also has shown limited efficacy for PBC, emphasizing the significant role of non-immunological pathology of epithelial cells in the pathogenesis.

This review will focus on pSS to illustrate the role of salivary gland epithelial cells (SGECs), looking for clues in viral infection, epigenetic dysregulation, structural disorder, secretory dysfunction, signaling pathways aberrations, homeostasis disruption, etc. We aim to offer novel perspectives, and cast a new light on future research into mechanisms and clinical treatment of pSS.

## Physiology of saliva secretion

Saliva serves numerous essential functions within the buccal cavity including flushing, bacteriostasis, and lubrication [37]. SGECs play a pivotal role of salivation [38], with acinar epithelial cells primarily responsible for secreting isotonic saliva, which is then modified by ducts and transported into the mouth. Surrounding ductal and acinar epithelium, myoepithelial cells contribute to the expulsion of saliva through contraction.

### Structure of SGECs

Epithelial cells are densely arranged and form the epithelium of SGs. SGECs exhibit polarization along the apical–basal axis, with the apical pole facing the lumen and the basal pole directed towards the deep connective tissue [39]. The apical membrane is equipped with microvilli and cilia to aid in mucus drainage. Additionally, aquaporins (AQPs), associated ion transporters and channels are also present in the apical membrane to facilitate fluid and electrolyte efflux. The basement membrane (BM) is located between the basal surface of the epithelium and the deep connective tissue. SGECs feature narrow cell gaps on the lateral sides, with extracellular matrix (ECM) in between, forming selectively permeable extracellular barriers between adjacent cells. The lateral membrane is tightly interconnected by cell junctions.

Cell junction is a specialized structure, which can be categorized into three groups: tight junctions (TJs), gap junctions (GJs), and anchoring junctions [40]. Anchoring junctions are further divided into desmosomes, hemidesmosomes (HDs), and adherens junctions (AJs). TJs serve as major intercellular junctions, forming barriers between epithelial cells essential for cell polarization and limiting paracellular permeability [41]. GJs facilitate intercellular communication and substance exchange. AJs and desmosomes reinforce cell adhesion, contributing to epithelial tissue integrity. HDs, located on the basal surface of the epithelium, connect the BM to the ECM. Cell junctions contribute substantially to cell polarity, which is important in directing secretion and absorption.

### Secretory process in SGECs

Ninety-nine percent of the final saliva is composed of water, with the remaining portion comprising various proteins and electrolytes. Salivation is a sophisticated process in SGECs. It is regulated by autonomic nerves, with neurotransmitters serving as the first messengers of salivary secretion, notably acetylcholine (ACh) and adrenaline [42].

#### Fluid and electrolyte secretion

In the main SGs, fluid and electrolyte secretion depend on cholinergic signals from the parasympathetic nerves [38]. ACh binds to the muscarinic receptors, initiating the activation of phospholipase C (PLC) and hydrolysis of phosphatidylinositol 4,5-bisphosphate (PIP2). PIP2 hydrolysis generates 1,4,5-triphosphate (IP3), which acts as the second messenger to trigger the elevation in intracellular Ca^2+^ concentration ([Ca^2+^]i) by binding to IP3R receptors (IP3Rs) [43]. As crucial Ca^2+^ channels within cells, IP3Rs mediate the rapid release of Ca^2+^ from endoplasmic reticulum (ER), and determine the initiation and pattern of Ca^2+^ signaling [44].

The Ca^2+^ signaling cascade is further amplified by store-operated calcium entry (SOCE). SOCE is mediated by Ca^2+^ release-activated Ca^2+^ (CRAC) channels, with STIM1, Orai1, and TRPC identified as pivotal components. STIM1 senses the consumption of Ca^2+^ in ER, leading to the interaction between STIM1 and Orai1, and subsequently recruiting TRPC channels. This process forms CRAC channels, enabling enormous Ca^2+^ influx and rapidly augmenting the elevation of [Ca^2+^]i [45].

The augmentation of Ca^2+^ signals ultimately trigger salivary fluid and electrolyte secretion. The steep rise in [Ca^2+^]i stimulates Ca^2+^-activated Cl^−^ channels ANO1 and K^+^ channels, and meanwhile evokes the transportation of AQP5 to the apical membrane [46]. Concurrently, during ion exchange, Na^+^/K^+^-ATPase hydrolysis sustains high intracellular K^+^ concentrations and extracellular Na^+^ concentrations, establishing a decreased Na^+^ chemical gradient inwardly [43]. As a result, water passes through paracellular transport or AQP5 into the lumen. Together with Cl^−^ channels on the apical membrane allowing Cl^−^ efflux, this process generates primary saliva highly enriched in NaCl is produced. Subsequently, in ductal cells, ion channels reabsorb Na^+^ and Cl^−^ while secreting small amounts of K^+^ and HCO3−, leaving the saliva hypotonic [47].

#### Protein secretion

Protein secretion is regulated by sympathetic nerves in SGs. The stimulation of β1-adrenergic receptor induces exocytosis of protein storage granules via the cAMP/PKA pathway [38]. During exocytosis, SNARE proteins play a crucial role in membrane trafficking and fusion [48]. Mucins, primarily MUC5B and MUC7 in human saliva, are the major proteins released through exocytosis [49]. Their main function is to preserve oral cavity moisture [50].

## Viral infection and SGECs

Viral infection and its possible contribution to pSS development have been studied for years. Currently, there is evidence suggesting that Epstein–Barr virus (EBV) and human T-cell leukemia virus type 1 (HTLV-1) infections are related to SGECs in pSS.

EBV has been most explicitly studied as a candidate directly inducing the phenotype discovered in pSS. EBV infects oral epithelial cells, replicates within them to produce progeny viral particles, and eventually leads to cell apoptosis and antigen release [51]. It was found that EBV-encoded small RNA binds to the SS autoantigen La/SSB, subsequently activating toll-like receptor (TLR)-3 and leading to production of proinflammatory cytokines [52]. Meanwhile, EBV can shuttle between epithelial cells and B cells, spreading the infection to B cells. As the lytic infection fades, a small amount of EBV can establish lifelong latent infection in memory B cells. This latent infection can be reactivated in response to environmental factors, and chronic or relapsing EBV infection in epithelial cells is associated with pSS [53]. It is recently established that EBV-specific microRNA (ebv-miR-BART13-3p) [54] exert their functions on SOCE, which will be discussed and illustrated in “The defect of calcium signaling” section and Fig. 2.

HTLV-I is believed to directly infect SGECs, causing alterations in cellular functions and inducing inflammation [55]. HTLV-1 virus particles are transmitted to SGECs through biofilm-like structures [56]. Infected cells produce molecules such as ICAM-1, IP-10, and RANTES, which are involved in adhesion, inflammation and migration [55, 57].

Although compelling evidence regarding viruses as environmental triggers for pSS is still lacking, their interactions with SGECs appear to partly mediate the emergence of the pSS phenotype.

## Epigenetic dysregulation in SGECs

Epigenetics encompasses heritable yet reversible alterations in phenotype not rooted in DNA sequence, including DNA methylation, non-coding RNAs, and histone modifications. In pSS, changes in DNA methylation and microRNAs are observed in SGECs.

### DNA methylation dysregulation

In SGECs from pSS patients, there is an overall decrease in DNA methylation level, contrasting with unaffected levels in T and B cells [58]. This suggests a preferential impact of epigenetic inheritance on epithelial cells. The DNA methylation deficit correlates with cytokeratin 19 [59] and ten-eleven translocation 2 (TET2) [60]. TET2 overexpression induces DNA hydroxymethylation, prompting demethylation associated with inflammation [60].

Consequently, SGEC dysfunction may partly result from altered DNA methylation level. For example, demethylation at the SSB gene promoter correlates with the overexpression of anti-SSB/La [61]. Moreover, the calcium pathway (involved in salivation, see details in “The defect of calcium signaling” section) experiences demethylation in SGECs, while the Wingless (Wnt) pathway (involved in cell survival and differentiation, see details in “Wnt and Hippo signaling pathway” section) undergoes methylation [62].

### MicroRNA dysregulation

Several studies reveal significant alterations in microRNA expressions in pSS in comparison to healthy controls, contributing to SGEC dysfunction and the emergence of immune characteristics. In one analysis, let-7b microRNA expression was downregulated in SGECs of autoantibody-positive pSS patients compared to negative patients [63]. Since let-7b microRNA can repress the transcriptional regulation of autoantigens SSA/Ro and SSB/La, its downregulation is implicated in their transcriptional dysregulation. Another study reported upregulation of miR-200b-3p in SGECs of pSS patients compared to sicca-complaining group, negatively correlating with Ro60/TROVE2 mRNAs in SGECs [64]. In an SS-susceptible mouse model, miR-146a expression was shown upregulated in the SGs at both 8 weeks (early phase of disease) and 20 weeks (late phase of disease) of age [65]. Further studies indicate that co-stimulatory molecule CD80 in SGECs is a potential target directly inhibited by miR-146a, thus altering CD86: CD80 ratio [66]. Moreover, miR-1248 [67] exerts its functions on SOCE, which will be discussed and illustrated in “The defect of calcium signaling” section and Fig. 2.

## Structural disorders of SGECs

**Fig. 1:**
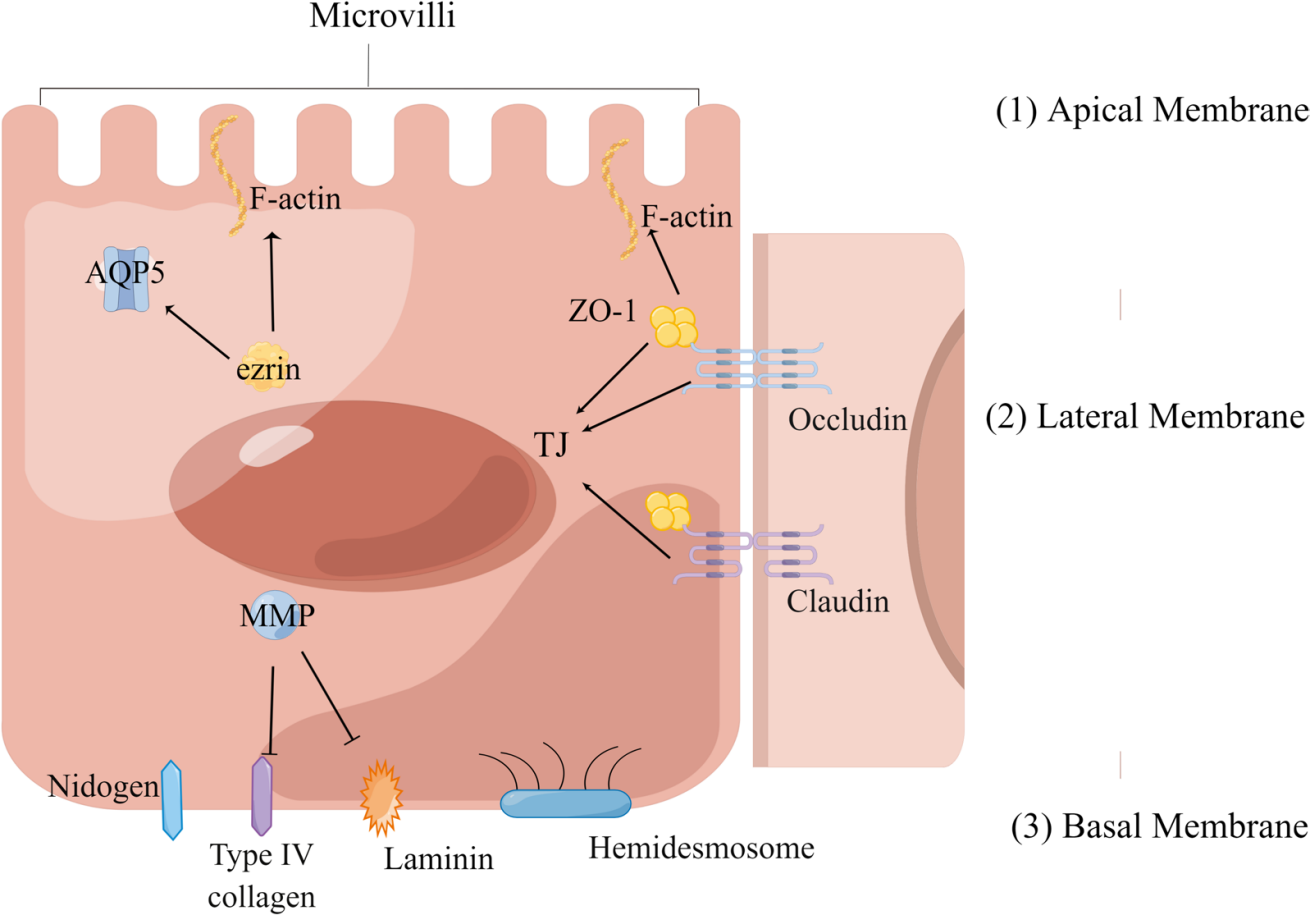
Ultrastructural changes of SGECs in pSS. (1) Changes in apical membrane: altered localization of ezrin might lead to redistribution of AQP5 and F-actin, the latter of which might contribute to morphological aberration of microvilli. (2) Changes in lateral membrane: altered expression of occludin, claudin and ZO-1 disrupts the structure of TJs. TJ disruption can also lead to loss of microvillus architecture because they are connected via F-actin. (3) Changes in basal membrane: the expression of laminin and nidogen is altered, and MMPs are over-activated to degrade Type IV collagen and laminin. Meanwhile, the structure of hemidesmosomes is also deteriorated. Abbreviations: aquaporin, AQP; tight junction, TJ; matrix metalloproteinase, MMP

The SG possesses a delicate structure that often faces disorder or even destruction in pSS patients. Multiple imaging techniques have frequently detected abnormal changes in SG tissue structure such as sialectasis, sparsity of the ductal branching pattern, and fat replacement [68–70]. The macrostructural changes in the SGs serve as indicators of microstructural changes in the epithelium. Evidence demonstrates that SGECs undergo considerable structural changes in their apical, lateral and basal structure as well as in the ECM, causing loss of barrier function and cell polarity, consequently resulting in dysfunction (see Fig. 1).

### Changes in the apical membrane

The alterations in the apical pole of SGECs mainly involve microvilli and AQP5, which share a common mechanism involving ezrin aberration.

An early study revealed a decrease in the number of microvilli on the apical surface, accompanied by a loss of their typical finger-shaped morphology [71]. Microvilli morphology is dependent on F-actin and is regulated by ezrin and its phosphorylated form. In control samples, ezrin was predominantly located in the apical area. In the acini of pSS patients, however, there is a significant increase in the expression of ezrin and P-ezrin, most of which is observed in the basal region rather than the apical pole. This shift in apical–basal cytoplasmic distribution [72] likely contributes to the disruption of the microvilli structure.

Regarding AQP5, its mislocation can be observed in acinar cells of pSS patients, which also seems to be partially associated with ezrin. In a study, researchers demonstrated protein–protein interactions between ezrin and AQP5, with their complexes found to be mislocalized to the basolateral region rather than the apical region in the acini of pSS [73].

### Changes in the lateral membrane

TJs on the lateral membrane are primarily engaged in the pathology of pSS. These TJs consist of both plasma membrane and cytosolic components. A notable decrease in the integrity of key proteins within this complex, such as occludins, ZO-1, claudins-1, -3 and -4, has been observed in SGECs affected by pSS [74]. At the genetic level, a study demonstrated apparent alteration in the genes encoding TJ proteins during the pre-autoimmune phase of the disease with phenotype resembling SS in C57BL/6.NOD-Aec1Aec2 mice. These genes include *TJP1*, *TJP2*, *AMOTL1*, *IGSF5*, *CLDN3*, *CLDN7*, *CLDN8* and *CLDN12* [75]. Such genetic disturbances suggest a disruption in the permeability of epithelial barriers, potentially leading to SGEC malfunction before immunoinflammatory responses. Of note, TJ disorder can trigger the loss of microvillus configuration, as microvilli and TJs are connected via F-actin [76].

### Changes in the BM

Labial salivary glands (LSGs) from pSS patients show tremendous changes in the BM, characterized by structural disorganization and loss of individual constituents [77]. Meanwhile, anoikis, a form of programmed cell death triggered by the separation of cells from ECM, has been identified in SGECs affected by pSS [78]. This phenomenon might be attributed largely to detachment of the epithelial cells and basal lamina [71]. Such events facilitate the invasion of inflammatory cells and trigger subsequent responses.

BM contains various components such as members of the laminin family, type IV collagen, and nidogens [79]. Laminin, the most abundant glycoprotein in BM, plays a role in maintaining epithelial cell polarity while mediating inflammation under certain circumstances [80]. Previous studies have reported overexpression of laminin-1 and -5, increased nidogen hydrolysis, and disturbances in the BM of SGs in pSS patients [77, 80–83]. Significant increases in both laminin mRNA and protein level were observed compared to normal controls, which took place devoid of periductal lymphocytic foci [80]. Elevated levels of laminin mRNA and protein, along with increased degradation of nidogens, indicated active remodeling of the BM. Such phenomena were less prominent in patients with a high degree of fibrosis and low salivary flow [81]. The BM disorganization might be partly attributed to the deteriorating effect of matrix metalloproteinases (MMPs). Previous studies have demonstrated enhanced activation of MMPs in acinar and ductal epithelial cells of LSGs from pSS, particularly showing an imbalance in the ratio of MMP-9 to its tissue inhibitor within the cytoplasm [71, 84]. As a consequence, the basal lamina becomes disorganized with degradation of laminin and type IV collagen.

In addition, cell–BM interactions such as HDs also contribute to basal structural disorder. Two main types of hemidesmosomes are identified in epithelia. Type I HDs consist of the integrin α6β4, protein BP180, CD151, BP230 and plectin, interacting with cytokeratin filaments, whereas type II HDs consist only of integrin α6β4 and plectin [85]. Alterations in these components are commonly observed in the SGs of pSS patients. Hypermethylation of the BP230 gene and an increase in BP230 protein level have been found in the acini of LSGs from pSS patients, while the BP180 protein level showed the opposite trend [86]. Meanwhile, α6β4 expression was observed not only in the basal laminae, but also in the cytoplasm and lateral plasma membrane [87]. Collectively, these results indicate substantial alterations in HDs and disorganization of the basal lamina, which may hinder ECM–cell communication.

In summary, the findings indicate that the early development of pSS is accompanied by regenerative processes of BM in SGECs. Immunoinflammatory involvement is facilitated due to structural disorder, which reciprocally causes more extensive changes of BM, resulting in anoikis of SGECs and glandular destruction.

### ECM remodeling

ECM is of great importance in cell survival, maintaining cellular structure and function, and mediating signal transductions [88, 89]. Physiological remodeling of ECM components (laminin and type IV collagen) depends on a delicate balance between proteolytic enzymes and their inhibitors [90]. Pronounced ECM destruction has been detected in the LSGs of pSS patients [88], while excessive or uncontrolled ECM destruction can disrupt the structure of the parenchymal cells and lead to severe tissue dysfunction [91]. Previous research has shown that the proteolytic activity of MMP-1, MMP-3 and MMP-9 on ECM macromolecules was significantly enhanced in LSGs extracted from SS patients. Interestingly, such changes occur concurrently with the loss of microvilli and disorders in BM, independent of the presence or the proximity to inflammatory cells [71, 88]. The aberrant degradation of ECM promotes the release of soluble damage associated molecular patterns (DAMPs), which can be recognized by B cells and activate Myd88-dependent signaling pathways. This may partly explain the underlying mechanism of B cell activation in pSS [92].

## Aberrations in salivary secretory process

Inadequate salivation is the culprit of xerostomia in pSS. Extensive research has been conducted over the decades to examine both fluid and protein secretion.

### Aberrations in fluid secretion

**Fig. 2:**
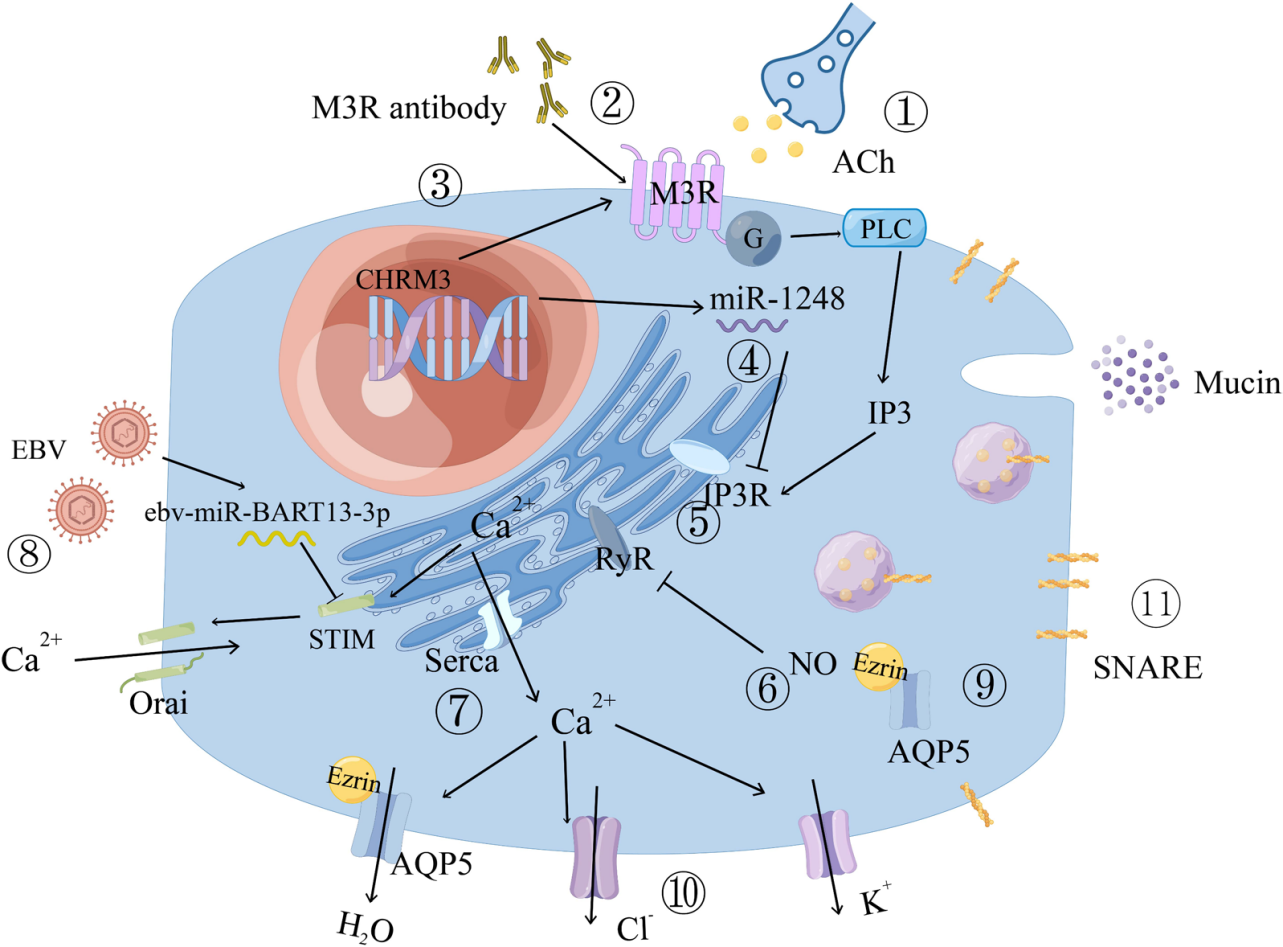
Aberrations in salivary secretory process. (1) Interfered neurotransmitter release; (2) M3R blockade/activation by antibody; (3) variants in M3R-related gene *CHRM3*; (4) inhibition of IP3R translation by the canonical function of miR-1248; (5) IP3R deficit; (6) reduction of RyR activity related to chronic NO elevation; (7) decreased expression of SERCA2b; (8) EBV-specific microRNA targeting at STIM1; (9) altered ezrin localization and AQP5 trafficking; (10) altered activity of apical ion channels; (11) SNARE redistribution and abnormal exocytosis of mucins. Abbreviation: M3R, M3 muscarinic ACh receptor; NO, nitric oxide; IP3R, 1,4,5-triphosphate receptor; RyR, ryanodine receptor; EBV, Epstein–Barr virus; AQP5, aquaporin 5

Given that water makes up 99% of saliva, aberrations of fluid secretion are considered to make utmost contribution to hyposalivation. The deviations may take place in the activation of muscarinic receptor, Ca^2+^ signaling, and apical channels (see Fig. 2).

#### The abnormal activation of muscarinic receptor

The initiation of signal transduction in the secretory process is attributed to the release of neurotransmitters and the activation of M3 muscarinic ACh receptor (M3R). Studies regarding innervation have shown no apparent loss of parasympathetic and sympathetic nerves, nor significant changes in distribution of immunoreactivity markers [93, 94], thus largely excluding the possibility of glandular denervation as a cause of secretory dysfunction in pSS. However, impaired neurotransmitter release or receptor binding has been observed in SGs. One study [95] showed that the acinar cells exhibited hyper-responsiveness to exogenous secretagogues, demonstrating their ability to function under certain stimulation, yet neurotransmitter release appeared to be somehow compromised. Another study suggested that it was the raised level of cholinesterase that mediated the metabolization of ACh, exerting an antisecretory effect in pSS [96].

In addition to neurotransmitters, malfunction of M3R represents another pathological factor. Upregulation of M3R in acinar cells, as demonstrated in one study [97], echoes with the hypersensitivity observed in the aforementioned experiment [95]. This up-regulation may result from impaired neurotransmission or blockade of M3R. Massive evidence has proved the existence of auto-antibodies in the serum of pSS patients targeting at extracellular loops of M3R [98–100]. By irreversibly binding to the second extracellular loop of M3R, most of these auto-antibodies act as antagonists of M3R. However, an increase in agonistic functional auto-antibodies targeting the first extracellular loop has also been detected [100]. The coordination and precise impact of these auto-antibodies on the hypofunction of SGs remain incompletely understood. Variants of the *CHRM3* gene, which encodes M3R, also appear to have potential associations with an increased risk of pSS, further highlighting the relevance of M3R in the pathogenesis of the disease [101].

#### The defect of calcium signaling

Subsequent to the signal transduction, inadequate Ca^2+^ signaling contributes substantially to the aberrations of secretory process in pSS. Studies have shown that acinar cells from pSS patients require a higher concentration of ACh to achieve the same Ca^2+^ mobilization [102], suggesting defects in Ca^2+^ signaling.

One important factor contributing to secretory dysfunction in Ca^2+^ signaling is the deficit of inositol trisphosphate receptors (IP3R). An enlightening study found that the acinar area unaffected by lymphocytic infiltration displayed a decrease in IP3R2 and IP3R3, without alterations in STIM1 or AQP5 [103]. This study unearthed early pathological changes in the gland, even before significant lymphocytic infiltration and tissue damage, can lead to hypofunction. Another study linked immune-dependent pathways with IP3R deficit through the dual functions of miR-1248 [67]. This microRNA inhibits the translation of IP3R and also induces interferon (IFN) production, resulting in suppression of Ca2+ signaling and stimulation of IFN signaling simultaneously.

Similar to IP3R, the ryanodine receptor (RyR) is also Ca^2+^-sensitive and modulates Ca^2+^ release from intracellular stores [104]. The regulation of RyR is influenced by cyclic adenosine diphosphate (cADP) ribose concentration, which is controlled by cyclic guanosine monophosphate (cGMP) [105]. Increased levels of nitric oxide (NO) have been observed in the serum of pSS patients [106], and NO participates in RyR modulation through the cGMP/ADPr/RyR pathway. The effects of NO on RyR activity depend on the duration of exposure [104]. Temporary elevation of NO leads to an increase in Ca^2+^ signal through the activation of RyR via cGMP. Prolonged elevation of NO, however, significantly reduces RyR activity independent of cGMP, probably through direct nitrosylation of RyR.

The aberration of SOCE holds significant importance in autoimmune diseases [141], and can cause the failure of Ca^2+^ signal augmentation. In the preliminary phase of pSS, a study demonstrated that an EBV-specific microRNA, ebv-miR-BART13-3p, targets the Ca^2+^ sensor STIM1, leading to attenuated SOCE [54]. This highlights the connection between viral infection and salivation loss, in which case immunoinflammatory responses are unnecessary.

Additionally, there have been reports of decreased expression of SERCA2b, a type of Ca^2+^ pump in ER, was also reported [107], although explicit evidence is limited.

Taken together, the evidence reveals that alterations in Ca^2+^ mobilization and subsequent hyposecretion are not necessarily consequences of immunoinflammatory responses.

#### The dysfunction of apical channels

The channels present in the apical membrane of SGECs, including ion channels and AQPs, serve as effectors of preceding pathways. Upon activation of muscarinic receptors and Ca^2+^ movement, the direct consequence is the translocation of AQPs to the apical membrane and an increase in ion channel activity [102, 108]. Theoretically, any pathological factors that interfere with neurotransmission and Ca^2+^ mobilization would influence ion transport and water permeability.

A common observation in various experiments is the mislocalization of AQPs due to erroneous trafficking. Instead of predominant distribution in apical membranes, AQP5 is remarkably expressed in basolateral membranes of SGECs of both model mice and human subjects with SS [109–112]. While there is controversy regarding whether AQP distribution has been altered [103, 113, 114], a significant body of evidence tends to support this phenomenon. Indeed, a majority of studies investigating ACh, M3R and Ca^2+^ mobilization mentioned above have observed AQP localization and acquired positive results.

Ions not only comprise the composition of saliva, but also serve as the driving force for water permeation. It was confirmed in acinar cells affected by pSS that the activation of Ca^2+^-dependent K^+^ channels and Cl^−^ channels is disrupted [102]. This disruption leads to a loss of the driving force for water efflux, consequently resulting in reduced salivary secretion.

### Aberrations in protein secretion

Abnormal protein secretion, particularly mucin exocytosis, may provide some rationale for the occurrence of pSS (see Fig. 2). It has been observed that in pSS patients without a significant decrease in salivary flow, the reduction of mucins in saliva can still cause severe xerostomia. This could be attributed to both the decreased quality of mucins and aberrations in the trafficking route [115]. Regarding the quality of mucins, it has been demonstrated that sulfotransferase Gal3ST activity markedly decreases in SGECs of pSS patients, resulting in hyposulfated mucins that are unable to retain moisture [116]. As for abnormal exocytosis, it is probably a consequence of sabotage of cellular structure. Exocytosis requires the participation of Rab family GTPases and SNARE proteins. In pSS patients, Rab3D and SNARE proteins such as VAMP8, syntaxin (STX) 3, STX4 and SNAP23 were relocated from the apical to the basal area of acinar cells, disrupting the cell polarity of SGECs. This leads to the aggregation of mucins in the cytoplasm and ECM rather than secretion into the duct [117, 118]; therefore, the amount of mucins in saliva is insufficient for lubrication and moisturization of the oral cavity. Moreover, the mis-distributed mucins (MUC5B and MUC7) mimic the role of DAMPs in stimulating the TLR4 signaling pathway and initiating inflammatory responses [119]. It should be noted that the compensatory increase in mucin synthesis in SGECs could exacerbate and perpetuate the inflammatory responses [120].

## Alterations of signaling pathways

As the center of the onset and progression of pSS, SGECs have undergone alterations in multiple signaling pathways to some extent. Previous studies have heavily emphasized immunological pathways such as NF-κB signaling, IFN pathways, TLR pathway, etc. [121]. Nevertheless, a few other pathways related to the functions of SGECs are gaining increased attention. Interestingly, abundant evidence suggests that these alterations are more deeply involved in the pathogenesis of pSS, with varying degrees of direct interrelationship with immunoinflammatory responses.

### Epidermal growth factor (EGF) signaling pathway

In the saliva of pSS patients, significantly low levels of EGF have been detected, which are related to the deterioration of saliva quality and severe intraoral manifestations [6]. An enhanced expression of the EGF/EGFR system, however, has been reported in SG samples extracted from pSS patients [122]. EGF is more than just a hormone secreted into exocrine fluid to stimulate growth, maintain functions, and defend against injury of gland epithelia. It can also be pro-inflammatory while anti-apoptotic, and may interfere with salivary secretion if it functions intracellularly.

EGF and its family play a complex and double-sided role in SGECs of pSS patients. They participate in the induction of inflammation in SGECs. The metalloproteinase ADAM17 promotes its family member amphiregulin to activate the EGF signaling pathway. As a result, extracellular signal-regulated kinase (ERK) 1/2 is phosphorylated to facilitate the release of inflammatory cytokines in pSS [123]. A few other studies, however, have suggested that EGF signaling could biologically function to prevent apoptosis in pSS. Stimulation of M3R by carbachol leads to the activation of EGFR mediated by Src, subsequently phosphorylating ERK and Akt pathways and suppressing apoptosis mediated by caspase 3/7 [124]. EGF was also proved to activate PI3K-Akt and NF-κB via distinct pathways to exert anti-apoptotic impact on SGECs [125].

### Transforming growth factor (TGF)-β signaling pathway

Epithelial–mesenchymal transition (EMT), a process whereby epithelial cells transition into a mesenchymal phenotype with inhibited expression of the epithelial marker E-cadherin and elevated vimentin expression, is recognized as the primary mechanism of fibrosis in SGs in pSS [126]. TGF-β has garnered increased attention in the study of pSS development due to its role in eliciting EMT and fibrosis in SGs. One study demonstrated an elevation of TGF-β1 protein in SG tissues in pSS compared to those of healthy controls [127]. The TGF-β signaling pathway can be triggered by TGF-β cytokine family, including bone morphogenetic protein (BMP). TGF-β or BMP binds to receptors, which phosphorylate SMAD2 and 3, or SMAD1, 5, and 8, forming complexes with SMAD4 that translocate into the nucleus. This leads to the activation of Snail, a transcriptional repressor of E-cadherin and a promotor of EMT [128]. In the non-canonical pathways of TGF-β, downstream factors such as RAS/RAF/MEK/ERK can also facilitate EMT in a SMAD-independent manner. Interestingly, IFN-γ/JAK/STAT pathway could activate SMAD7, known as the “anti-SMADs”, to repress TGF-β signaling pathway [129]. This may implicate an unconventional perspective on the role of the TGF-β and IFN-γ/JAK/STAT pathway, since JAK inhibitors are traditionally considered therapy for pSS patients yet with ambiguous efficacy.

Among the TGF-β superfamily, BMP might provide promising prospect for pathogenesis study and therapy development for pSS. Analysis of LSG epithelia from eight pSS patients and eight non-SS controls by RNA sequencing revealed upregulation of BMP related genes [130]. Another study also confirmed that BMP-6 increased in SGs of pSS patients and possibly induced SG hypofunction irrespective of immune activation and autoantibody production [131]. The increase in BMP-6 is correlated with the overexpression of lysosome-associated membrane protein (LAMP)-3, lysosomal exocytosis of heat shock protein 70, and subsequently induced TLR4 signaling [132, 133].

### Wnt and Hippo signaling pathway

The Wnt signaling pathway contributes to cell proliferation and differentiation during SG development [134]. In pSS, studies have shown alterations in the expression levels of key components of this pathway. For instance, Wnt1 and Wnt3a expression levels were found to be elevated in the SG of pSS patients, while inhibitors of the pathway such as Dickkopf-related protein (DKK)-1 and sclerostin exhibited decreased levels in the serum [135]. Additionally, genetic polymorphism studies have identified several risk genes of pSS within the canonical Wnt/β-catenin pathway, including *LRP5*, *FRZB*, and *ADIPOQ* [136]. Adiponectin (ADIPOQ), in particular, acts as a regulator of the Wnt/β-catenin pathway and has been associated with anti-inflammatory effects, potentially through the induction of interleukin (IL)-10 secretion [137]. Notably, SG epithelial cells from pSS patients have shown increased expression and secretion of ADIPOQ compared to healthy subjects [138].

Similarly, the Hippo signaling pathway, known for its role in regulating organ size, cell proliferation, and differentiation, is also involved in SG development [139]. In pSS, dysregulation of this pathway leads to a shift in its effects from cell maintenance to destruction. Rather than interacting with E-cadherin to maintain cell polarity and junctional integrity, the downstream effectors such as TAZ have been demonstrated to localize in the nucleus and act as transcriptional factors due to the inhibition of the upstream kinase Lats2 [140]. This mislocalization results in the accumulation of ECM components such as fibronectin and connective tissue growth factor, potentially contributing to ECM remodeling and tissue fibrosis. Importantly, the structural defect caused by Hippo signaling dysfunction has been linked to hypo-salivation, independent of lymphocytic infiltration.

In summary, both the Wnt and Hippo signaling pathways play significant roles in SG development and are dysregulated in pSS, leading to pathological changes such as inflammation and tissue fibrosis.

## Homeostasis disruption

**Fig. 3:**
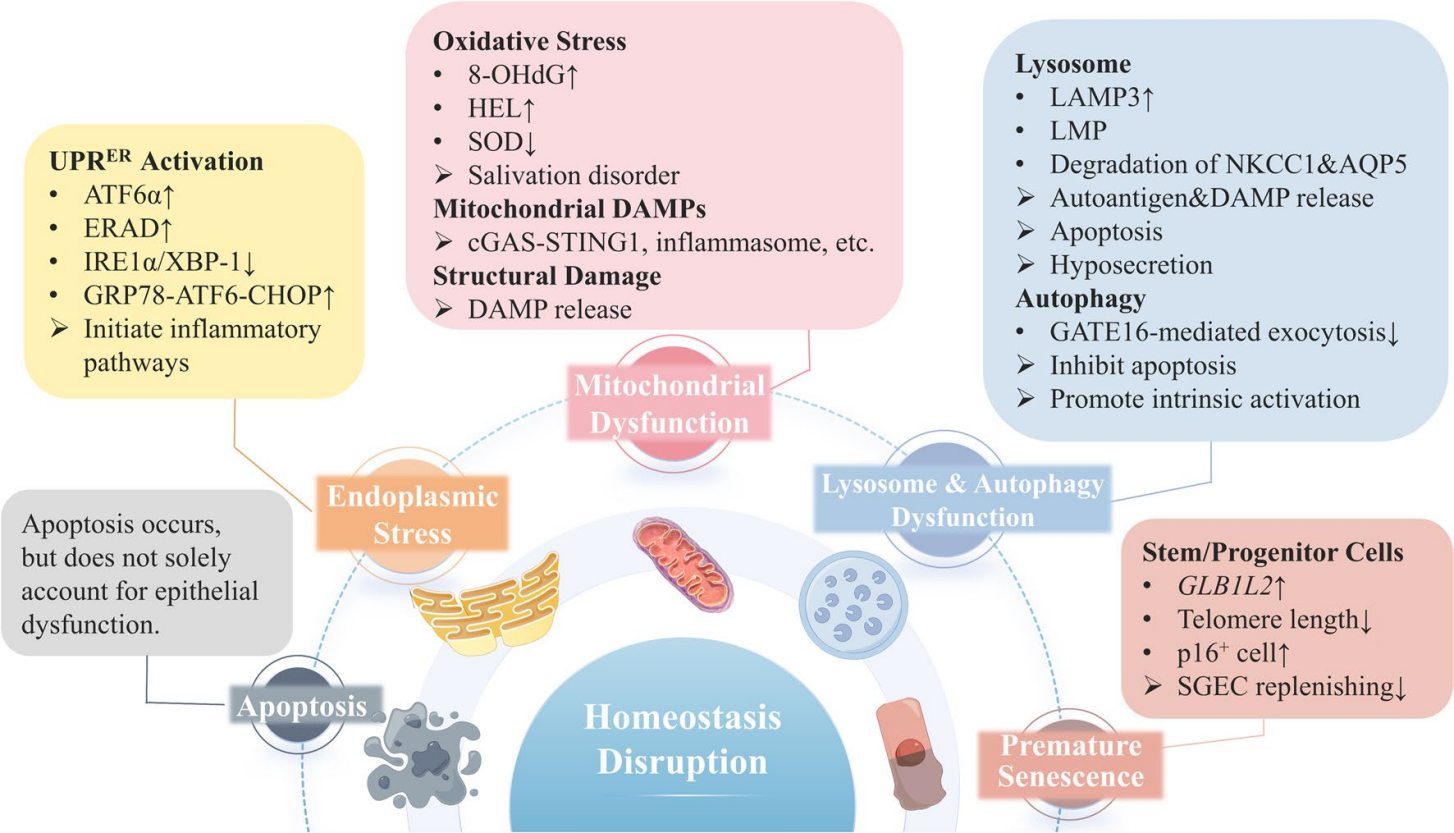
Homeostasis disruption in SGECs. The homeostasis disruption in SGECs include apoptosis, endoplasmic stress, mitochondrial dysfunction, lysosome and autophagy dysfunction, and premature senescence of stem/progenitor cells. In the box, the dots represent manifestations, and the arrows represent consequences. *UPR* unfolded protein response, *IRE* inositol-requiring enzyme, *ATF* activating transcription factor, *ERAD* ER-associated protein degradation, *GRP* glucose-regulated protein, *HEL* hexanoyl-lysine, *LMP* lysosomal membrane permeabilization

The perturbations of cellular homeostasis can lead to cellular dysfunction, phenotypic alterations, cellular senescence, and even cell death, which may be important factors in the diminished biological viability and heightened immune response observed in SGECs (see Fig. 3).

### The apoptosis of SGECs

Traditionally, impaired SG function in pSS has been attributed to epithelial apoptosis. Existing research suggests that apoptosis of SGECs in pSS is mediated by a multitude of mechanisms, including Fas-mediated pathways [141–143], organellar dysfunction [133, 144–147], CD8^+^T lymphocyte [148], B lymphocytes and autoantibodies [149–151], SSA/Ro52 autoantigen [152, 153], cytokines [154–156], TLRs signaling [157–159], STAT3 signaling [160, 161], and other pathways [162–167].

According to the conventional viewpoint, pSS patients with low salivary flow are expected to have minimal functional salivary tissue. However, investigations have shown a low frequency of apoptosis in SGECs from pSS patients, with no clear association between apoptosis incidence and foci score [168]. Similar findings have been replicated in animal models [169]. Additionally, a genome-wide association study revealed significant upregulation of anti-apoptosis genes in SGECs of pSS patients [170].

The hypothesis that epithelial apoptosis predominantly causes glandular damage is not entirely consistent with current research findings. It is more plausible that while epithelial cell apoptosis occurs in pSS, it alone may not fully account for the marked reduction in salivary secretion.

### The endoplasmic stress in SGECs

ER serves crucial functions especially in regulating protein homeostasis by overseeing various processes including protein biogenesis, folding, assembly, transport and degradation. Excessive protein secretion or perturbed ER protein folding can lead to aggregation of unfolded or misfolded proteins within the ER lumen, which is termed as ER stress, a condition that activates the unfolded protein response (UPR^ER^). UPR^ER^ is sensed by three ER transmembrane stress receptors: inositol-requiring enzyme 1α (IRE1α), activating transcription factor 6α (ATF6α) and PKR-like endoplasmic reticulum kinase [171]. Activation of UPR^ER^ aims to reduce translation to alleviate the burden on nascent protein folding, degrading unfolded proteins, and increasing chaperone expression to facilitate protein folding.

Cells with high secretory requirements, such as acinar cells of the SGs, are particularly susceptible to environmental factors that impose ER stress. Exposure to pro-inflammatory stimulant such as reactive oxygen species (ROS), cytokines and DAMPs can elicit UPR^ER^, while alterations in UPR^ER^ signaling can reciprocally initiate inflammatory pathways [172].

Studies have shown that the SGs of pSS patients experience persistent ER stress. Growth arrest and DNA damage inducible protein 153, a canonical marker of ER stress response, displayed elevated expression in the ductal epithelium of NOD mice and pSS patients [173]. Increased ATF6α pathway and ER-associated protein degradation (ERAD) activities were observed in LSGs from pSS patients, potentially contributing to preventing apoptosis [174]. Additionally, IRE1α/XBP-1 pathway, another branch of UPR^ER^, has been found impaired in LSGs of pSS patients, with heightened methylation levels of their promoters [175]. ERdj5, critical in ERAD for processing misfolded proteins, was found upregulated in the LSGs of pSS patients, while *ERdj5*−/− mice exhibited SS-like phenotypes, including decreased SG function, periductal inflammation in SGs and elevated serum autoantibodies levels [176]. Moreover, abnormalities in protein secretion can induce ER stress, as evidenced by the overexpression and aggregation of MUC1 in the ER of acinar epithelial cells from pSS patients, along with its co-precipitation with glucose-regulated protein 78 (GRP78) [177]. A recent study found that the GRP78-ATF6-CHOP signaling was hyperactive in LSGs of pSS patients, and suppression of this pathway increased salivation in a murine model [147], implicating ER stress in SG lesions in pSS.

### The mitochondrial dysfunction of SGECs

Mitochondria, essential in energy production, apoptosis, Ca^2+^ homeostasis, and innate immunity, are the major source of ROS in the process of generating energy through oxidative phosphorylation [178]. When ROS production exceeds the capacity of antioxidant protection, oxidative stress (OS) takes place.

Several studies have implicated OS in the injury of SGs in pSS patients. Two OS markers, 8-hydroxy-2ʹ-deoxyguanosine (8-OHdG) and hexanoyl-lysine (HEL), were proved to be increased in the saliva of pSS patients, whereas such changes were absent in individuals with SG dysfunction unrelated to pSS or in healthy controls. Moreover, the mitochondrial glutamic-oxaloacetic transaminase level in the saliva of pSS patients was remarkably correlated with the 8-OHdG and HEL levels [179]. A knockout mouse with a deletion of superoxide dismutase related gene was reported to have reduced salivary secretion without obvious inflammation of the SGs [180], suggesting a potential role of OS in salivary secretion disorders.

Dysfunctional mitochondria not only generate high levels of ROS, but also appear to serve as important inflammatory triggers. Considering that mitochondria are believed to have originated from symbiotic aerobic prokaryotic bacteria, various components and metabolic byproducts of mitochondria can function as DAMPs, thereby promoting inflammation upon release into the cytoplasm or extracellular milieu [181]. These mitochondrial DAMPs can activate several signaling pathways, including the cyclic GMP-AMP synthase and stimulator of IFN response cGAMP interactor 1 (cGAS-STING1) signal pathway, inflammasome signal pathway, and inflammatory pathways mediated by pattern recognition receptors [181]. These mechanisms may contribute to the intrinsic activation observed in SGECs of pSS patients [182, 183].

Evidence of structural mitochondrial damage in epithelial cells derived from pSS patients has been documented. Transmission electron microscopy revealed mitochondrial membrane disruption, cristae loss and disorganization, and mitochondrial matrix swelling in SGECs of pSS patients [184–186]. Given the pivotal role mitochondria play in various cellular processes, abnormalities in mitochondrial structure and function inevitably result in impaired epithelial function.

### The lysosome and autophagy dysfunction of SGECs

Lysosomes are membrane-bound cellular organelles containing digestive enzymes that have been found to participate in various cellular processes, including secretion, biomolecule degradation, immune response, cell death, etc. Through autophagy, a self-catabolic process, lysosomes dispose of intracellular contents [187].

Lysosomal abnormalities within SGECs can contribute to the development of pSS through several mechanisms. The LAMP3 have been found expressed higher in SGECs of pSS patients in comparison to control groups. This overexpression of LAMP3 was thought to be related with the release of autoantigens including components of SSA (TRIM21), SSB (La), and α-fodrin protein via extracellular vesicles, independently of apoptosis mechanisms [146]. Animal experiments have demonstrated that altered lysosomal function, via LAMP3 overexpression in SGECs, was involved in the salivary dysfunction and autoimmune activation [133]. Lysosomal damage often results in lysosomal membrane permeabilization (LMP) [188], leading to the inappropriate release of cathepsin into the cytoplasm, which in turn triggers caspase-dependent apoptosis of SGECs [189]. Dysfunctional lysosomes may exacerbate the degradation of membrane proteins crucial for salivation, such as NKCC1 and AQP5 [190]. Additionally, increased lysosomal exocytosis leads to the release of DAMPs, which activate immune cells via TLRs receptors [132].

Autophagy is a cellular degradation process that eliminates futile or impaired components through lysosome-based mechanisms. Autophagy is important in maintaining cellular homeostasis and is conducive to cell survival. In one study involving human SGEC lines, ER stress inducers were able to elicit autophagy, which subsequently inhibited apoptosis following ER stress in SGECs [145]. Notably, autophagy-deficient mice exhibited an SS-like phenotype characterized by immune cell infiltration in exocrine tissues, and impaired GATE16-mediated exocytosis in SG acinar cells has been found [191]. The aforementioned studies underscore the protective role of autophagy in SGECs during glandular damage. A recent study, however, discovered that autophagy in SGECs of pSS patients was correlated with histologic disease severity. Stimulation of human SGEC lines with peripheral mononuclear cells and serum from pSS patients induced autophagy and release of adhesion molecules, which could be reversed by autophagy inhibitor [192]. This suggests that autophagy may play a role in the intrinsic activation of SGECs.

### Premature senescence of stem/progenitor cells

The preservation of SG hinges notably on the delicate balance between cell death and proliferation modulated by resident mature stem/progenitor cells [193]. Stem cells possess the capability for unlimited self-renewal, giving rise to progenitor cells that subsequently lose this capacity. Their distinction between these cell types, however, remains somewhat ambiguous, leading to the common usage of the term “stem/progenitor cell” [194, 195]. In the context of normal SG homeostasis, salivary gland progenitor cells (SGPCs) proliferate and differentiate into new epithelial cells, replenishing those lost due to damage or senescence [196–198].

In pSS, the premature senescence of stem/progenitor cells within SGs has garnered increasing attention. One study presented evidence of premature aging in salivary gland stem cells (SGSCs) among pSS patients [199], wherein both the quantity and self-renewal capacity of SGSCs were significantly diminished compared to healthy controls. This premature senescence phenomenon manifests at various stages of the disease progression. In the early stage of pSS, prior to lymphocyte focus formation, an upregulation of beta-galactosidase-like gene *GLB1L2*, considered a hallmark of cellular aging, was observed. In later phases, STELA analysis showed a sharp reduction in the telomere length in SGSCs from pSS samples, indicative of extensive replicative history [199].

Beyond SGSCs, senescence in SGPCs is also demonstrated in early stages of pSS. As well established, cell senescence is marked by characteristic expression of p16, a suppressor of cell division kinase 4 [200]. Compared to healthy controls, SGs of pSS patients generally exhibit a higher prevalence of p16^+^ cells within both the epithelium and the SGPC niche [201]. This phenomenon is associated with defective SG secretory function, but does not correlate with systematic disease activity. Moreover, individuals exhibiting signs suggestive of potential development into pSS, albeit not meeting the ACR-EULAR criteria, exhibit an elevated number of p16^+^ cells within epithelium and SGPC niche compared to non-SS sicca patients, whereas at levels lower than observed in confirmed pSS patients. This denotes an early and continuous process in the pathogenesis of pSS [201].

Intriguingly, senescent cells can develop a senescence-associated secretory phenotype, characterized by the secretion of various growth modulators, angiogenic factors, pro-inflammatory cytokines, MMPs, etc. [202]. The existence of this phenotype within SGs of pSS patients, and its impact on stem/progenitor cells, warrants further investigation.

## Conclusions and future prospect

Epithelial lesions in autoimmune epithelitis, including reduced salivation observed in pSS, are complicated phenomena that cannot be solely attributed to lymphocytic infiltration. Alterations in SGECs such as viral infection, epigenetic dysregulation, structural disorder, secretory dysfunction, signaling aberrations, and homeostasis disruption all have a role in the occurrence and development of pSS (see Table 1). It is noteworthy that some of these alterations can occur independently of or in absence of lymphocytic infiltration, and may even serve as upstream factors for immuno-inflammatory responses. Given the limited efficacy of current immunological treatments, we reckon these findings significant for re-examining the pathogenesis of pSS and developing interventions targeting SGECs in the early stages of the disease.

**Table 1:** Changes in SGECs, their significance and consequences

Specific changes in salivary gland epithelial cells (SGECs)	Significance	Consequences	References
Epstein–Barr virus (EBV) infection	Production of progeny viral particles	Resulting in cell apoptosis and antigen release	[51]
EBV infection	Binding autoantigen La/SSB	Induce inflammation	[52]
EBV infection	Inhibiting components of Ca^2+^ release-activated Ca^2+^ (CRAC) channels	Result in hyposecretion	[54]
Human T-cell leukemia virus type 1 (HTLV-1) infection	Production of adhesion molecules	Induce inflammation	[55, 57]
Defective DNA methylation at the SSB gene promoter P1	Overexpression of anti-SSB/La	Induce autoimmunity	[61]
Demethylation of calcium pathway	Aberrations in calcium pathway	Result in hyposecretion	[62]
Methylation of Wingless (Wnt) pathway	Activation of Wnt pathway	Promote survival and differentiation	[62]
Down-regulation of microRNA let-7b	Overexpression of anti-SSA/Ro and anti-SSB/La	Induce autoimmunity	[63]
Upregulation of miR-200b-3p	Negative correlation with Ro60/TROVE2 mRNAs	Related with induction of autoimmunity	[64]
Upregulation of miR-146a	Alteration of CD86: CD80 ratio	Regulate immunity	[65, 66]
Upregulation of miR-1248	Inhibit expression of IP3R	Result in hyposecretion	[67]
Upregulation of miR-1248	Induce secretion of interferon (IFN)	Induce inflammation	[67]
Ezrin re-distribution	Microvilli structure disruption	Decrease cell surface area	[71, 72]
Ezrin re-distribution	Aquaporin (AQP) mislocation to the basolateral region	Result in hyposecretion	[73]
Tight junction (TJ) disruption	Disturbance in the permeability of epithelial barriers	Result in malfunction of SGECs	[74, 75]
Altered laminin expression, nidogen hydrolysis	Basement membrane (BM) remodeling	Result in structural disorder and anoikis	[77, 80–83]
Enhanced activation of matrix metalloproteinases (MMPs)	Degradation of BM components	Result in structural disorder and anoikis	[71, 84]
Alteration of hemidesmosomes (HDs)	Disrupted cell-BM interactions	Hinder extracellular matrix (ECM) -cell communication	[86, 87]
Enhanced activation of MMPs	Degradation of ECM components and release of damage associated molecular patterns (DAMPs)	Induce lymphocytic infiltration/autoimmunity	[71, 88, 91]
Impaired neurotransmitter release	Decreased activation of M3 muscarinic ACh receptor (M3R)	Result in hyposecretion	[95, 96]
M3R antibody	M3R activation or blockade	Result in aberrant secretion	[98–100]
Variants of M3R-related gene	M3R malfunction	Result in hyposecretion	[97, 101]
Deficit of IP3R	Defective calcium signaling	Result in hyposecretion	[67, 103]
Reduced ryanodine receptor (RyR) activity	Defective calcium signaling	Result in hyposecretion	[104]
Decreased expression of SERCA2b	Defective calcium signaling	Result in hyposecretion	[107]
Altered AQP and ion channels	Defective ion transport and water permeability	Result in hyposecretion	[102, 109–112]
Decreased activity of sulfotransferase	Decreased quality of mucins	Result in inadequate moisture preservation	[116]
Abnormal exocytosis of mucins	Insufficient mucin secretion	Result in inadequate moisture preservation	[117, 118]
Abnormal exocytosis of mucins	Accumulation of mucins in ECM	Induce inflammation	[119]
Alterations in epidermal growth factor (EGF) signaling	Release of inflammatory cytokines	Induce inflammation	[123]
Alterations in EGF signaling	Inhibition of apoptosis	Maintain cell viability	[124, 125]
Alterations in transforming growth factor (TGF)-β signaling	Induction of epithelial–mesenchymal transition (EMT)	Induce fibrosis	[127, 128]
Alterations in TGF-β signaling	Increase in bone morphogenetic protein (BMP)-6	Result in hyposecretion	[130, 131]
Alterations in Wnt signaling	Promotion of proliferation and survival, inhibition of inflammation	Maintain cell viability	[135, 136, 138]
Alterations in Hippo signaling	ECM remodeling	Induce fibrosis	[140]
Alterations in unfolded protein response (UPR^ER^) signaling	Chronic endoplasmic reticulum (ER) stress	Induce inflammation	[173, 175–177]
Alterations in UPR^ER^ signaling	Inhibition of apoptosis	Maintain cell viability	[174]
Alterations in UPR^ER^ signaling	Induction of apoptosis	Decrease cell viability	[147]
Mitochondria dysfunction	Occurrence of oxidative stress	Result in hyposecretion	[179, 180]
Structural damage of mitochondria	Release of mitochondrial DAMPs	Induce inflammation	[184–186]
Lysosome dysfunction	Release of autoantigen and DAMPs	Induce lymphocytic infiltration/autoimmunity	[132, 146]
Lysosome dysfunction	Induction of apoptosis	Decrease cell viability	[189]
Lysosome dysfunction	Additional degradation of membrane proteins involved in salivation	Result in hyposecretion	[190]
Increased autophagy	Inhibition of apoptosis	Maintain cell viability	[145, 192]
Increased autophagy	Promoting the expression of adhesion molecules	Induce lymphocytic infiltration/autoimmunity	[192]
Defects in autophagy	Impairment of exocytosis	Result in hyposecretion	[191]
Upregulation of *GLB1L2* and telomere length reduction in salivary gland stem cells (SGSCs)	Premature senescence of stem/progenitor cells	Lack of replenishment of fresh SGECs	[199]
Elevated expression of p16 in salivary gland progenitor cells (SGPCs)	Premature senescence of stem/progenitor cells	Lack of replenishment of fresh SGECs	[200, 201]

Several non-immunological strategies show promising prospects for ameliorating or reversing epithelial lesions and treating xerostomia in pSS. For example, muscarinic agonists, such as pilocarpine [203], can effectively enhance secretion by promoting calcium-induced trafficking of AQP to the apical membrane. Low-intensity pulsed ultrasounds [204] and gene delivery of *hAqp1* [205] have also demonstrated the ability to elevate AQP expression. Bone marrow therapy and spleen cell therapy [206] can replenish stem cells to restore damaged epithelial cells. Furthermore, potential intervention targets include ZO-1 and claudins, which regulate TJ structure; MMPs, which influence BM and ECM; EGF, which promotes epidermal healing and cross-influences neurotransmission; SMADs and BMP6, which participate in TGF-β signaling and fibrosis; and UPR^ER^ signaling, which induce apoptosis. However, a more comprehensive understanding of the pathological mechanisms is necessary for successful translation into clinical therapies.

## Data Availability

Not applicable.

## Abbreviations

pSS: Primary Sjögren’s syndrome
SG: Salivary gland
PBC: Primary biliary cholangitis
SGEC: Salivary gland epithelial cell
AQP: Aquaporins
BM: Basement membrane
ECM: Extracellular matrix
TJ: Tight junction
GJ: Gap junctions
AJ: Adherens junctions
HD: Hemidesmosomes
ACh: Acetylcholine
PLC: Phospholipase C
PIP2: Phosphatidylinositol 4,5-bisphosphate
IP3(R): 1,4,5-Triphosphate (receptor)
[Ca2+]i: Intracellular Ca2+ concentration
ER: Endoplasmic reticulum
SOCE: Store-operated calcium entry
CRAC: Ca2+ release-activated Ca2+
EBV: Epstein–Barr virus
HTLV-1: Human T-cell leukemia virus type 1
TLR: Toll-like receptor
TET2: Ten-eleven translocation 2
Wnt: Wingless
P-ezrin: Thr-567 Phosphorylated ezrin
LSG: Labial salivary glands
MMP: Matrix metalloproteinase
DAMP: Damage associated molecular patterns
M3R: M3 Muscarinic ACh receptor
IFN: Interferon
RyR: Ryanodine receptor
cADP: Cyclic adenosine diphosphate
cGMP: Cyclic guanosine monophosphate
NO: Nitric oxide
STX: Syntaxin
EGF(R): Epidermal growth factor (receptor)
ERK: Extracellular signal-regulated kinase
TGF: Transforming growth factor
EMT: Epithelial–mesenchymal transition
BMP: Bone morphogenetic protein
LAMP: Lysosome-associated membrane protein
LRP: Lipoprotein receptor-related protein
FRZB: Frizzled-related protein
DKK: Dickkopf-related protein
ADIPOQ: Adiponectin
IL: Interleukin
UPR: Unfolded protein response
IRE: Inositol-requiring enzyme
ATF: Activating transcription factor
ROS: Reactive oxygen species
GADD: Growth arrest and DNA damage inducible protein
ERAD: ER-associated protein degradation
GRP: Glucose-regulated protein
OS: Oxidative stress
8-OHdG: 8-Hydroxy-2ʹ-deoxyguanosine
HEL: Hexanoyl-lysine
LMP: Lysosomal membrane permeabilization
SGPC: Salivary gland progenitor cell
SGSC: Salivary gland stem cells
